# Camp Life for Consumptives

**Published:** 1901-11

**Authors:** C. H. Wilkinson

**Affiliations:** Twenty-six years Chief Medical Officer and Surgeon to St. Mary’s Hospital, Galveston, Texas


For Texas Medical Journal.

### Camp Life for Consumptives.

BY C. H. WILKINSON, M. D.,
Twenty-six years Chief Medical Officer and Surgeon to St. Mary's Hospital, Galveston. Texas.

Perhaps no treatment has, in modern times, undergone more evolution than that of phthisis. Perhaps no disease in medical nosology, on account of the vast range of its baneful influence, has deserved more evolution in its treatment, than has consumption. Certainly, the nature and pathology of none have undergone more radical interpretation during the past century than those of the disease under present consideration. It is not, however, the intention of the writer to enter into either the nature or pathology of tuberculosis in the present article. These have been fixed and determined through the investigations of Koch, and are now beyond the range of speculation or controversy, and through the researches of this illustrious investigator it may be said that the first gleam of rational treatment can be seen on the horizon of medical progress.

The trend of this article will be in the direction of a better and more satisfactory method of dealing with consumption in the future. Any one who has attempted to keep pace with the therapeutical department of phthisis for the past twenty years must have been struck with the vast array of remedies and systems, and even specifics, that have been heralded over the universe for the arrest of tubercular disease of the lungs. It would be a waste of time to enumerate them. Nearly every physician is familiar with them, and it will suffice to say that probably more money has been gathered in, more quackery has been displayed through, and more disappointments have resulted from these various remedies than in the case of any other disease that ever existed.

Today, the list of remedies vaunted for the cure of phthisis is legion, and scarcely does a week elapse without a new one being added to the number.

The strangest fact connected with these medical discoveries is that temporary benefit results from the employment of nearly every one of them; the surest fact resulting from an investigation into their therapeutic value is that few, if any of them, ever produced a permanent recovery.

It has been the fortune of the writer to come in contact with very many cases of consumption, and he has had an excellent opportunity of testing many varieties of treatment, and for watching the results in others. He therefore reiterates the statement that, while the majority of remedies lately employed in that disease will afford a temporary relief, few if any of them can be relied upon for cures. This experience can no doubt be corroborated by the majority of physicians who have had an extensive dealing with consumption.

Thus it would appear that, in order to secure more satisfactory results in the treatment of tuberculosis of the lungs, we must, for the present at least, turn away from the pharmacopœa and the usual modern plans of treatment, and seek through other means the object so devoutly wished.

Not that drugs, however, are useless in the treatment of consumption; on the contrary, as adjuvants they are very valuable, and for the relief of many symptoms they are indispensable. It is probably not going too far to make the broad assertion here that the day is not far distant when medicine will control tuberculosis as effectually as mercury and quinine now control the subjects of their respective domains.

Such, however, is not the case today, and pending the arrival of so fortuitous an epoch it behooves us to investigate a field which beyond all doubt is *proven,* and which, if properly regarded and taken advantage of, will reverse the statistics just stated by restoring to health the vast majority of its seekers, with a minimum of unsatisfactory results upon the other hand.

A review of authorities upon the subject of consumption, for the past fifty years, reveals a unanimity of opinion amongst them in regard to the great value of "open air" for cases of phthisis. Nearly every writer upon that disease commends a change of air, be it to the mountain top, or a trip to sea, experience having demonstrated that more proportion of benefited cases resulted through such trips and changes than through any other agency that the profession of medicine was able to offer. Thus, the great value of climate in phthisis came to be an established fact, and hence it is that the "open air treatment" for that disease is recognized today by all familiar with the subject as the most reliable, the most satisfactory, and the most hope-inspiring method of dealing with consumption.

This question is no longer one of theory, but of facts. I have too much evidence of the wonders of a suitable climate for the benefit of phthisis, to doubt its efficacy. Much of this testimony I have accumulated through personal observation and inspection, covering a period of many years, and I do not hesitate to say that, if the people of the United States only understood the marvelous advantages of a suitable atmosphere for consumptive cases to live in, if they could see the cures produced by a prolonged sojourn in such a climate, as I have seen, then the western portion of the State of Texas, the ideal sanitarium of the world, would in the next few years become as populous as Massachusetts, and the Mecca for that great army of pilgrims always marching over other routes to the inevitable "bourne whence no traveler e'er returns."

It will be readily seen from the foregoing that the writer believes that, in the climate of Western Texas, we have the ideal resort for cases of consumption. In fact, instances by the hundreds could be collected in illustration of the soundness of his faith. Too late has this knowledge been held from the public, and hence the specific object of this paper will be devoted to what I conceive to be the proper place and the proper plan for taking care of our phthisical cases in the near future.

Western Texas constitutes all that portion of the great Lone Star State lying between longitudes 96 and 107. Its boundaries are the Colorado river on the east and the Rio Grande on the west, the 32 degree of north latitude on the north and the Gulf of Mexico on the south. Inside these boundaries may properly be styled the natural sanitarium of the world. The surface of this area is undulating, and rises gradually from its eastern boundary, from an elevation of 500, to its western limits, where it attains an elevation of 5000 feet. It is traversed by four great systems of railroads, and is studded with numerous cities and flourishing villages. The atmosphere there is dry and pure, so much so, that it is the boast of its inhabitants, in its central portion, that fresh meat hung up in a shady place in summer never taints; and I have heard it said that wounds inflicted, under ordinary care, never suppurate. The days are warm in summer, but the nights are always cool and clear; an abundance of crystal water can be found in the springs and streams abounding there, and the luxuries of life can easily be obtained at most of the stopping places on the railroads. Thus, it would appear that Western Texas furnishes all that is necessary to satisfy the demands of phthisical patients.

The Plan or Method of Treating Consumptives.—The method which I would impress upon the reader is the "camp life," or "open air" plan of treatment for the cure of pulmonary tuberculosis, or consumption. The experience of all authorities upon the subject favors it, while that of the writer certainly emphasizes the great and lasting value of "open air." It is well known that soldiers while in camp rarely ever contract consumption, although exposed to all the vicissitudes of weather, but on the contrary, they become hardier and more rugged by reason of such life. Taking this observation as a guide in handling consumptives, I would suggest the camp, the bivouac, for such cases. Establish such camps in any of the numerous eligible places to be found in Western Texas. Pitch your tents near railroad cities where modern comforts might be obtained, and maintain such order and morale over your inmates as the vast importance of the undertaking demands. In such camp life your cases get the maximum of fresh air, one of the greatest objects to be desired; but besides these, they secure that discipline so imperatively needed in the management of congregated cases, and besides, the watchful care at all times of a competent physician. To all such camps sufficient ground should be attached for purposes of exercise, while gardens, fields and orchards might be cultivated by those of the soldiery who might wish to avail themselves of such a privilege, thereby combining pleasure and profit in the pursuit of health. A camp maintained along these lines would meet the greatest requirement of phthisical patients, and in the course of a few years the success attending such a method would be followed by the establishment of others, and thus in the course of time the mountains and prairies of West Texas would, like those of Colorado, be dotted with hamlets and villages of consumptives restored to health, where lately before, the cowboy and his herd of cattle dominated the land.

The plan just briefly outlined is not an expensive one, although as a matter of course some capital would be required in order to perfect a thoroughly equipped encampment. But no other plan known to the writer carries with it so many of the essentials for a successful undertaking as this does, for the outlay required.

We have in the first place a carefully selected atmosphere and elevation; a mode of living for our cases where the greatest amount of pure and fresh air is always available; a system of discipline and hygiene always to be maintained, without which the treatment of congregated cases of consumptives could never be successful; and lastly, we have at all times over them the watchful eye of some competent physician. Auxiliary to the general plan of living, I suggest that remunerative employment about the place be given cases, when available, as it is a well known fact that, in order to derive the permanent benefits sure to be obtained by following the "out door" plan of treatment, patients should remain subject to the above mentioned environments for months, if not for years. This prolonged sojourn is an expense to the masses, and tends to defeat the object sought after by compelling many to return to their homes before being cured, thereby bringing on relapses.

Employment about the camp means, not only some remuneration, but a pleasant pastime for the patient, and the increase in valuation of the place. It is the means of keeping those cases in the proper atmosphere until they are well; for be it known, the greatest offset to the successful management of "resort" consumptives is their too often premature return from healthy places to former unhealthful and unsuitable homes.

When we consider that there are today in the United States over 100,000 cases of consumption, most of whom are amenable to treatment if properly cared for, and that no adequate facilities are afforded to the majority of them for the relief of their condition; and when we further consider the melancholy fact that the average "health resort," usually frequented by such patients, returns unsatisfactory dividends to the statistics of tuberculosis, because their "stopping places" are so ignorantly managed and so poorly maintained, then we ought to see at a glance that in the proper handling of consumptives, after the method I have laid down, not only is the greatest hope held out to the invalid with phthisis, but a rich reward, financially, is in sight for him who enters into such an undertaking.

